# Combined Treatment with Low-Dose Ionizing Radiation and Ketamine Induces Adverse Changes in CA1 Neuronal Structure in Male Murine Hippocampi

**DOI:** 10.3390/ijms20236103

**Published:** 2019-12-03

**Authors:** Daniela Hladik, Sonja Buratovic, Christine Von Toerne, Omid Azimzadeh, Prabal Subedi, Jos Philipp, Stefanie Winkler, Annette Feuchtinger, Elenore Samson, Stefanie M. Hauck, Bo Stenerlöw, Per Eriksson, Michael J. Atkinson, Soile Tapio

**Affiliations:** 1 Institute of Radiation Biology, Helmholtz Zentrum München, German Research Center for Environmental Health GmbH (HMGU), 85764 Neuherberg, Germany; daniela.hladik@helmholtz-muenchen.de (D.H.); prabal.subedi@helmholtz-muenchen.de (P.S.); jos.philipp@helmholtz-muenchen.de (J.P.); stefanie.winkler@helmholtz-muenchen.de (S.W.); atkinson@helmholtz-muenchen.de (M.J.A.); 2 Chair of Radiation Biology, Technical University Munich (TUM), 80333 Munich, Germany; 3 Department of Environmental Toxicology, University Uppsala, 75236 Uppsala, Sweden; sonja.buratovic@ebc.uu.se (S.B.); per.eriksson@ebc.uu.se (P.E.); 4 Research Unit Protein Science, Helmholtz Zentrum München, German Research Center for Environmental Health GmbH (HMGU), 80939 Munich, Germanyhauck@helmholtz-muenchen.de (S.M.H.); 5 Research Unit Analytical Pathology, Helmholtz Zentrum München, German Research Center for Environmental Health GmbH (HMGU), 85764 Neuherberg, Germany; annette.feuchtinger@helmholtz-muenchen.de (A.F.); samson@helmholtz-muenchen.de (E.S.); 6 Department of Immunology, Genetics and Pathology, Uppsala University, 75185 Uppsala, Sweden; bo.stenerlow@igp.uu.se

**Keywords:** hippocampus, proteomics, BDNF, CA1 neurons, dendrite abnormality, Golgi staining, irradiation

## Abstract

In children, ketamine sedation is often used during radiological procedures. Combined exposure of ketamine and radiation at doses that alone did not affect learning and memory induced permanent cognitive impairment in mice. The aim of this study was to elucidate the mechanism behind this adverse outcome. Neonatal male NMRI mice were administered ketamine (7.5 mg kg^−1^) and irradiated (whole-body, 100 mGy or 200 mGy, ^137^Cs) one hour after ketamine exposure on postnatal day 10. The control mice were injected with saline and sham-irradiated. The hippocampi were analyzed using label-free proteomics, immunoblotting, and Golgi staining of CA1 neurons six months after treatment. Mice co-exposed to ketamine and low-dose radiation showed alterations in hippocampal proteins related to neuronal shaping and synaptic plasticity. The expression of brain-derived neurotrophic factor, activity-regulated cytoskeleton-associated protein, and postsynaptic density protein 95 were significantly altered only after the combined treatment (100 mGy or 200 mGy combined with ketamine, respectively). Increased numbers of basal dendrites and branching were observed only after the co-exposure, thereby constituting a possible reason for the displayed alterations in behavior. These data suggest that the risk of radiation-induced neurotoxic effects in the pediatric population may be underestimated if based only on the radiation dose.

## 1. Introduction

Ionizing radiation is an integral part of medical treatment and diagnostics. In radiotherapy, healthy tissues outside the target volume are exposed to low-dose radiation. Therefore, the possibility of radiation-associated risks must be considered. This is especially so for children, since exposure to low doses of ionizing radiation is associated with increased risk of malignancies and cognitive impairment later in life [1,2,3]. 

In paediatric radiotherapy and imaging, sedation is often applied during imaging to ensure immobilization [4,5]. Ketamine exerts seductive properties via blockage of the N-methyl-D-aspartate (NMDA) receptors in the brain. These ionotropic glutamate receptors are involved in synaptic plasticity, learning, and memory [6,7,8]. Ketamine exposure during early brain development has been shown to induce neurodegeneration, followed by cognitive impairment [9,10,11]. 

The effects of combined exposure to clinically relevant doses of ketamine (7.5 mg kg^−1^ body weight) and whole-body low-dose radiation (100 mGy, 200 mGy) during brain development were studied in mice [12]. The co-exposed mice showed lack of habituation, hyperactivity, and reduced learning and memory capabilities, while mice exposed to single agents showed no significant differences in behavior compared to non-exposed controls. A combination of drug and irradiation consistently impaired cognitive function [12]. 

The aim of this study was to elucidate molecular mechanisms that could be associated with the previously observed cognitional impairment. Therefore, we used mice that were treated identically to the previous experiment [12]. We found that the combination of low-dose radiation and ketamine consistently changed the hippocampal proteome. The combination treatment altered the structure of CA1 neurons, while individual treatments did not display this effect. Neuronal morphology, such as dendrite complexity and spin density, are strongly correlated with neuronal function. Therefore, these observations provide a plausible mechanistic reasoning for the detrimental interaction of ketamine and radiation in the developing brain of newborn mammals.

## 2. Results

### 2.1. Analysis of the Hippocampal Proteome after Single or Combined Treatment 

**Figure 1:**
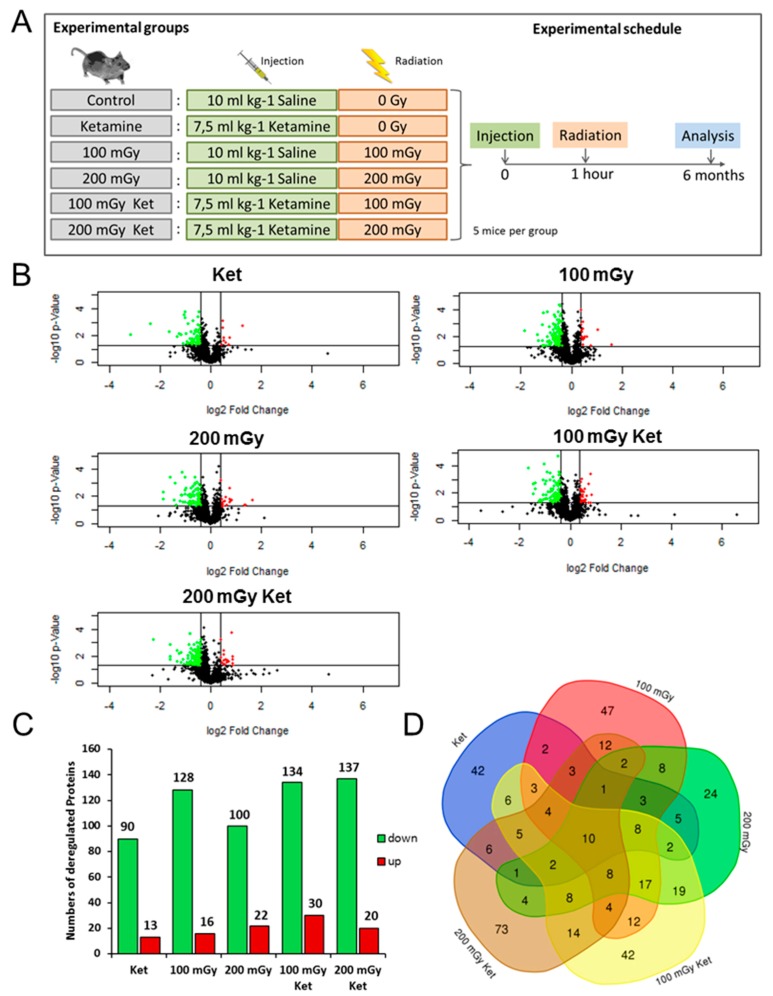
Changes in the hippocampal proteome after single treatment or co-treatment. (**A**) Schematic presentation of the experimental groups and the treatment schedule. (**B**) Volcano plots representing the distribution of all quantified proteins (identification with at least two UP) in hippocampi exposed to single treatment with ketamine (Ket), gamma radiation (100 mGy, 200 mGy), or combined treatment (100 mGy Ket, 200 mGy Ket). Deregulated proteins (*p* ≤ 0.05, fold-change ±1.3) are highlighted in green (downregulated) and red (upregulated). (**C**) Total numbers of significantly downregulated (green) and upregulated (red) proteins are shown for all treatments (*p* ≤ 0.05, fold-change ±1.3). (**D**) Venn diagram illustrating the number of shared deregulated proteins between the five experimental groups.

The hippocampal proteomes of all of the mice were analyzed using label-free LC/MS-MS. In total, 2668 proteins were identified, of which 1839 were quantified based on at least two unique peptides (UP) (Tables S1–S5). Volcano plots of all quantified proteins showed the distribution of nonregulated and deregulated proteins (Figure 1B). Using the filtering criteria (identification with two UP, *p* ≤ 0.05, fold-change ±1.3) the analysis showed the following numbers of significantly deregulated proteins in comparison to the control group: 103 in the ketamine (Ket) group, 144 in the 100 mGy group, 122 in the 200 mGy group, 164 in the 100 mGy Ket group, and 157 in the 200 mGy Ket group (Figure 1C). Shared deregulated proteins in the different experimental groups are shown in the Venn diagram in Figure 1D. The two co-exposure groups shared 55 deregulated proteins (Figure 1D). These are listed alongside the fold-changes and GO biological functions in Table 1. The majority of these proteins were classified as members of actin cytoskeleton organization or neuronal development.

### 2.2. Effects on Neuronal Cytoskeleton and Synaptic Plasticity Following Combined Exposure to Ketamine and Irradiation 

**Figure 2:**
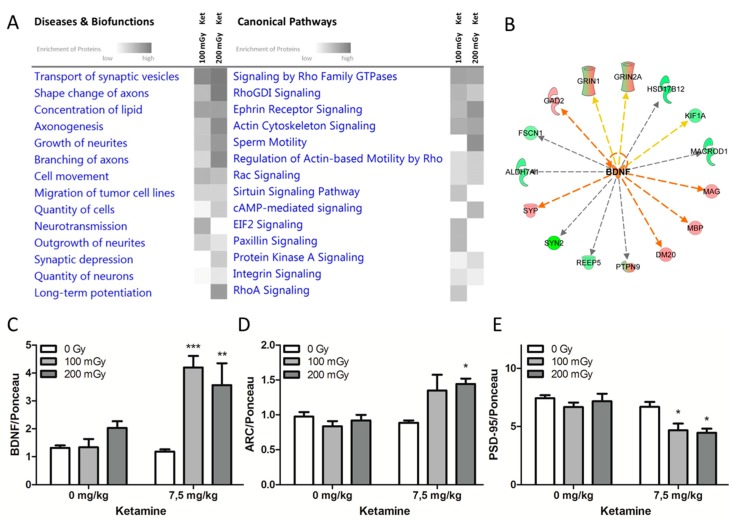
Combined treatment with ketamine radiation affected neuronal morphology and synaptic plasticity. (**A**) The Ingenuity Pathway Analysis (IPA) of associated signaling pathways based on all significantly deregulated hippocampal proteins in the co-exposure groups is shown. The functions and pathways in the categories “canonical pathways” and “diseases and biofunctions”, respectively, were ranked by their significance and displayed using a gray color gradient (the darker the color, the higher the pathway score). The pathway scores represent the negative log of the *p*-value derived from the Fisher′s exact test, where all gray boxes have a *p*-value of ≤0.05; n = 5. (**B**) Prediction of activation of brain-derived neurotrophic factor (BDNF) (orange color) was based on the deregulated proteins from the co-exposure groups. The upregulated proteins are marked in red and the downregulated proteins are in green. Immunoblot analyses of the relative expression of (**C**) BDNF, (**D**) ARC, and (**E**) PSD-95 in single and combined treatment groups were normalized to the total amount of proteins measured by Ponceau staining (Figure S1). Error bars represent the SEM, n = 4, * *p* < 0.05, ** *p* < 0.01, *** *p* < 0.001 (two-way ANOVA with Bonferroni multiple testing).

To better understand the involvement of biological processes following the combined treatment with ketamine and radiation, the 55 common significantly deregulated proteins from the co-exposure groups were subjected to Ingenuity Pathway Analysis (IPA). In particular, the categories “canonical pathways” and “diseases and biofunctions” were analyzed (Figure 2A). The most enriched canonical pathways were involved either in the organization of the cytoskeleton (signaling by Rho GDI family GTPases, RHOGDI signaling, actin cytoskeleton signaling, Rac signaling, RhoA signaling, regulation of actin-based motility by Rho) or played a role in neuronal transmission (ephrin receptor signaling, cAMP-mediated signaling, integrin signaling). Similarly, the most affected biofunctions were related to reorganization of the neuronal structure (shape change of axons, axonogenesis, growth of neurites, branching of axons) and synaptic transmission (transport of synaptic vesicles, neurotransmission, synaptic depression, long-term potentiation) (Figure 2A).

Activation of the brain-derived neurotrophic factor (BDNF) was predicted based on the deregulation profiles of the co-exposed groups by IPA (Figure 2B). BDNF is one of the key regulators of neuronal morphology and stimulates the growth and differentiation of new neurons and synapses [13]. In good agreement with this, the level of BDNF investigated by immunoblotting showed a significant increase in its expression in the co-exposed groups (upregulation by mean fold-changes of 4.2 (*p* < 0.001) and 3.6 (*p* < 0.01) in the groups “100 mGy Ket” and “200 mGy Ket”, respectively) (Figure 2C). 

To further investigate the possible activation of BDNF in the co-exposed groups, the expression of a downstream target of BDNF, the activity-regulated cytoskeleton-associated (ARC) protein, was measured. In agreement with the upregulation of BDNF, the expression level of ARC was significantly increased in the group that received ketamine and gamma radiation (200 mGy, *p* < 0.05) (Figure 2D). In addition to BDNF, postsynaptic density protein 95 (PSD 95) influences both synapses and neuronal branching. [14] Only the combined treatment with ketamine and radiation caused a significant reduction in the level of PSD-95 (*p* < 0.05) (Figure 2E). Ponceau stainings and Western blot bands are shown in Figure S1.

### 2.3. Morphological Abnormalities of Hippocampal CA1 Neurons only after Combined Treatment with Ketamine and Irradiation

**Figure 3:**
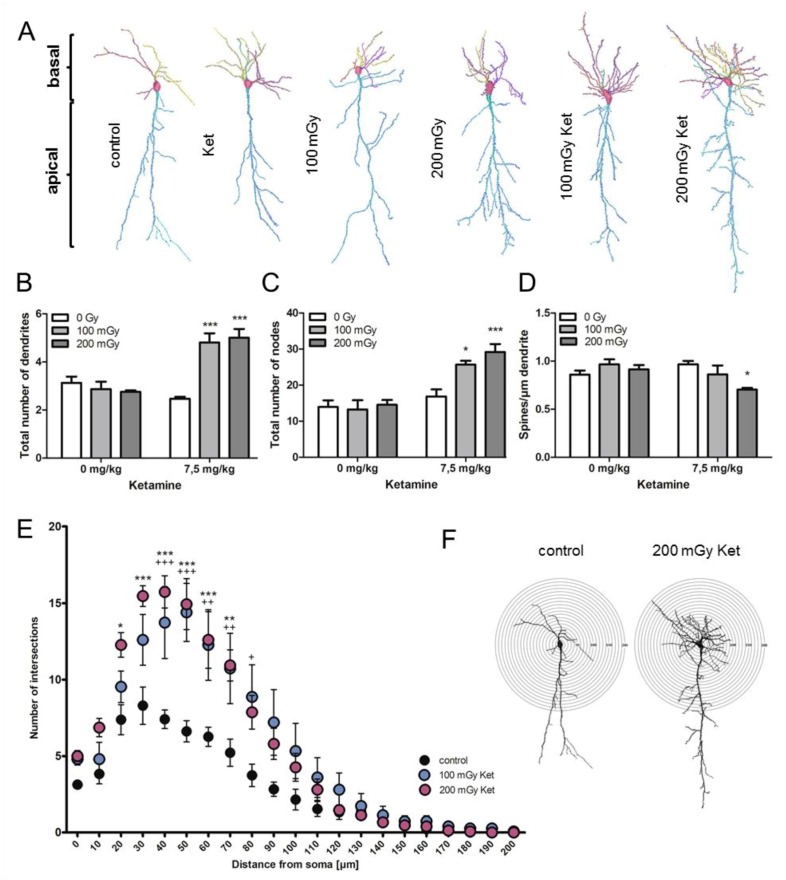
Co-treatment with ketamine and irradiation led to structural changes in hippocampal CA1 neurons. (**A**) Reconstructed hippocampal CA1 neurons representative for each experimental treatment group are shown. Each individual dendrite is presented in a different color. (**B**) The number of basal dendrites is shown in all experimental groups. Five neurons per animal with 5 biological replicates were analyzed; *** *p* < 0.001. (**C**) The number of nodes representing the branching points in basal dendrites is shown. Five neurons per animal with 5 biological replicates were analyzed; * *p* < 0.05, *** *p* < 0.001. (**D**) The spine densities of the basal dendrites are shown. Five neurons per animal with 5 biological replicates were analyzed; * *p* < 0.05. (**E**) A comparison between the total number of dendritic intersections for each circle between the controls (black) and the co-treated neurons (100 mGy Ket: blue; 200 mGy Ket: purple) was performed. The co-treated animals showed significant increase in the number of intersections between 40 and 80 µm (100 mGy ket; + *p* < 0.05, ++ *p* < 0.01, +++ *p* < 0.01) and between 20 and 70 µm (200 mGy Ket; * *p* < 0.05, ** *p* < 0.01, *** *p* < 0.001). At least 5 neurons per animal with 5 biological replicates were analyzed. The first values (2 µm circle) represent the total number of basal dendrites, as shown in (**B**). (**F**) Representative CA1 neurons with concentric circles used for the Sholl analysis are shown. The radius interval between the circles was set to 10 µm per step, ranging from 10 to 200 µm from the center of the neuronal soma to the end of the dendrites. The numbers of dendritic intersections per circle were quantified. At least 5 neurons were analyzed per animal. The *p* values were calculated using a two-way ANOVA with Bonferroni multiple testing.

Golgi-Cox staining followed by dendritic reconstruction was performed on tissue sections of the Cornu Ammonis (CA1). Raw images are presented exemplarily for every experimental condition in Figure S2. Representative images of reconstructed neurons are shown in Figure 3A. Apical and basal dendrites were analyzed separately in all experimental groups (Figure 3B). No effect was found on the structure and number of apical parts of the CA1 neurons (Figure S3A–C). A significant increase (*p* < 0.001) in the total number of basal dendrites was present after the combined treatment with ketamine and radiation (Figure 3B). In the co-treated groups, the total number of nodes was significantly increased (100 mGy Ket: *p* < 0.05, 200 mGy Ket: *p* < 0.001) while there was no difference in the single treatment groups compared to the control (Figure 3C). The number of spines divided by the dendrite length was significantly reduced in the group co-exposed to 200 mGy Ket (*p* < 0.05) (Figure 3D). However, the reduction of spines was not significant in the 100 mGy Ket group and therefore could not explain the observed cognitional impairment. No effect in spine number was detected in the apical dendrites (Figure S3C). This indicated an increase in the complexity of the basal dendrites after co-exposure. 

To investigate this in more detail, Sholl analysis, representing the distribution of dendritic intersections with increasing distance from the cell soma, was performed. A significant shift in the number of intersections in the circle diameter of 20–80 µm was observed in the co-exposed groups compared to the sham-treated controls, thereby confirming a significant increase in the number of basal dendrites and their branching points (Figure 3E,F). 

## 3. Discussion

Pediatric radiotherapy and treatment frequently require ketamine sedation prior to irradiation [15]. Concerns about the long-term safety of this combination have been raised following the report of cognitive impairment in mice co-exposed to clinically relevant doses of ketamine and irradiation [12]. We show here that co-exposure during early brain development results in persistent alterations to both the proteome and structure of hippocampal CA1 neurons. Significant increases in dendrite number and branching were observed when ketamine was given immediately prior to irradiation at 100 or 200 mGy. Neither ketamine nor radiation treatment alone induced the reorganization of dendritic structures. 

The structure of dendrites has a profound impact on the processing of neuronal information, including learning and memory. The formation of the dendritic arbour, the sides of synaptic connections from input neurons, is usually completed by adulthood. Aberrations and remodeling of dendritic structures are observed only under pathological conditions [16,17,18]. Extension of the dendrite length beyond the normal level is related to mental retardation [19,20]. Neuropathic pain linked to depression and cognitive decline is known to be associated with an increase in dendritic length and branching [21]. 

Interestingly, ketamine (10 μM) was previously shown to promote both the number of dendritic branches and the total length of the arbours in embryonic rat cortical neurons in vitro [22] due to ketamine-induced rapid increase in BDNF secretion [23,24]. Cranial irradiation of adult mice using radiation doses higher than in this study (1 or 10 Gy) resulted in significant reductions in dendritic branching and total length in the hippocampus [25]. Our data showed increased basal branching of CA1 neurons with the combined exposure, therefore suggesting a radiation-enhanced ketamine-like response in the neuronal structure and demonstrating the strong impact of ketamine and irradiation when applied together.

In contrast, the regulation of neuronal structures is dependent upon several factors. Small GTP binding proteins, like Ras-related C3 botulinum toxin substrate 1 (RAC1), Ras homolog gene family member A (RHOA), and cell division control protein 42 homolog (CDC42), are crucial for reorganization of the dendrites and their branching [26,27,28,29]. In accordance with this, our proteome analysis showed that most of the significantly deregulated proteins in the co-exposed groups belonged to pathways dependent on the Rho family GTPases. Thus, changes in the neuronal cytoskeleton and associated pathways (shape change of axons, axonogenesis, growth of neurites, and branching of axons) were all predicted from the proteome data and were consistent with the changes in CA1 neuronal morphology. Secreted factors such as neurotrophins are known to play key roles in regulating dendrite outgrowth and branching [30]. BDNF is a well-studied mediator of synaptic plasticity and memory formation [31,32]. Overexpression of BDNF in CA1 neurons was shown to improve fear and object-location memory in mice [33], but was associated with cognitive impairment in MECP2-duplication syndrome [34]. Application of exogenous BDNF was shown to increase the number of dendrites in pyramidal neurons [35]. Ketamine given at doses of 10 or 15 mg kg^−1^ (but not at 5 mg kg^−1^) resulted in a rapid and significant increase in the expression of hippocampal BDNF in adult male Wistar rats [36]. In contrast, high-dose cranial irradiation (10 Gy) of adult C57BL/6 mice resulted in a significant decrease in BDNF expression in the hippocampus one month after exposure [37]. In our study, only the co-treatment with ketamine (7.5 mg kg^−1^) and low-dose radiation (100 mGy or 200 mGy) led to an increase in the total amount of BDNF in the hippocampus. This increase was sustained long after exposure in neonatal mice. 

Similar to BDNF, PSD-95 is involved in synaptic functions, especially with regard to NMDA receptors [38,39]. We previously showed that whole-body irradiation of neonatal NMRI or C57BL/6J mice causes increased expression of PSD-95 in the hippocampus six months after exposure [40,41]. This was seen at whole-body doses equal to or higher than 0.5 Gy, but not at lower doses. Similarly, it was shown that high-dose (1 Gy, 10 Gy) cranial gamma-radiation caused increased expression of PSD-95 in the hippocampus of adult mice 30 days after irradiation [25]. Administration of high-dose ketamine (30 mg kg^−1^ over five consecutive days) was shown to immediately increase the level of PSD-95 in the synaptosomes of adult male Wistar rats [42]. Lower doses of ketamine (10 mg kg^−1^) did not produce effects on the total level of PSD-95 in the hippocampal membranes of mice immediately (30 min) after administration [43]. In addition to synaptic functions, both BDNF and PSD-95 play a role in regulating dendrite outgrowth and branching [14,35]. In contrast to BDNF, PSD-95 inhibits the branching of dendrites [14]. Contrary to our previous results obtained using a less-sensitive slot blotting method [12], the immunoblotting used here showed reduced expression of PSD-95 after the combined treatment with ketamine and low-dose radiation. No change in the PSD-95 level was detected using single exposure treatments. The persistent upregulation of BDNF and downregulation of PSD-95 after the co-exposure, in addition to the changes in the expression levels of several other proteins responsible for neuronal growth and branching (CDC42, PAK3, ARF6, and others displayed in Table 1), could be the molecular explanation for the observed increase in the number and branching of basal dendrites in the CA1 neurons.

## 4. Materials and Methods 

### 4.1. Animals

All procedures were in accordance with the European Communities Council Directive of 24 November 1986 (86/609/EEC; approval date: 26 April 2013) after permission by local ethical committees (Uppsala University) and the Swedish Committee for Ethical Experiments on Laboratory Animals. The results were reported in line with relevant aspects of the ARRIVE guidelines [44].

Pregnant Naval Medical Research Institute (NMRI) mice were purchased from Scanbur (Sollentuna, Sweden) and housed in Makrolon^®^ III cages. Only the male offspring were used in the experiments to mimic the conditions used for cognitional testing [12].

### 4.2. Exposure

Neonatal (postnatal day 10) mice were exposed to a single subcutaneous injection of ketamine (7.5 mg kg^−1^ body weight) or to a low dose of whole-body gamma radiation (^137^Cs; 100 mGy, 200 mGy), or co-exposed. In the co-exposure group, ketamine was administered one hour before irradiation. Control mice were injected with 10 mL kg^−1^ body weight saline (0.9%) and sham-irradiated. Control mice and mice irradiated only were not given sedatives prior to exposure. The dosages of ketamine and irradiation were determined based on the previous experiments showing no effect of single exposures on spontaneous behavior, learning and memory, or protein levels [12,45,46,47]. A schematic presentation of the experimental design is shown in Figure 1A.

### 4.3. Tissue Collection

The mice were sacrificed with CO_2_ 6 months post-treatment. Brains were excised, dissected, and rinsed in cold PBS. The right hemisphere was used for proteome analysis. The hippocampi were microdissected, snap frozen in liquid nitrogen, and stored at −80 °C. The left hemisphere was used for the morphology study. 

### 4.4. Protein Lysis and Determination of Protein Concentration

Frozen hippocampi were pulverized and suspended in RIPA buffer (Thermo Fischer, Darmstadt, Germany) enriched with phosphatase and protease inhibitors (Sigma-Aldrich, Taufkirchen, Germany).

After sonication, lysis, and centrifugation, protein concentrations were measured using BCA Protein Assay Kit (Thermo Fischer) according to the manufacturer’s instructions. 

### 4.5. Mass Spectrometry (MS) 

Label-free measurements were performed on a QExactive high field mass spectrometer (Thermo Fisher) in data-dependent acquisition mode, as described previously [48,49]. 

### 4.6. Protein Identification and Quantification

Spectra were analyzed using Progenesis QI software (Version 3.0, Nonlinear Dynamics) for label-free quantification, as described before [48]. The filtering criteria were as follows: Proteins identified and quantified with two UP and fold-changes of ≤0.77 or ≥1.3 (*t*-test; *p* ≤ 0.05) were considered to be significantly differentially expressed.

### 4.7. Pathway Analysis 

The list of significantly deregulated proteins with their accession numbers, fold-changes and *p*-values were imported into Ingenuity Pathway Analysis (IPA, QIAGEN Redwood City, www.qiagen.com/ingenuity). 

### 4.8. Western Blot Analysis

Western blots were performed according to the protocol described previously [48]. The following antibodies were used: BDNF (abcam, ab203573, Cambridge, UK), ARC (abcam, ab118929, Cambridge, UK) and PSD-95 (abcam, ab18258, Cambridge, UK). Ponceau (Sigma-Aldrich, St. Louis, MO, USA) staining served as an internal loading control (Figure S1). 

### 4.9. Golgi Staining

Staining was performed using the FD Rapid GolgiStain Kit (NeuroTechnologies, Columbia, SC, USA) according to the user manual. Directly after dissection, the hemispheres were put into 5 mL Golgi-Cox solution for fixation and impregnation for one week and then frozen at −20 °C. For imaging, the frozen brain was cut with a cryostat at −20 °C (100 µm coronal sections), and the sections were mounted on gelatin-coated microscope slides.

### 4.10. Imaging and Analysis of Dendrites and Spines

CA1 neurons were reconstructed using Neurolucida software (MBF Bioscience, Williston, ND, USA) under 40x magnification (Zeiss AxioPlan 2 microscope) on 100 µM coronal sections and analyzed with Neurolucida Explorer software (MBF Bioscience). Apical and basal dendrites were separately analyzed. In the Sholl analysis, the radius interval of each section was set to 10 µm, starting from 10 µm and ending at a 200 µm distance from the soma. 

### 4.11. Statistical Analysis

Statistical analysis of the LC-MS/MS data was performed with Excel using a two-sided Student’s t-test. The Western blotting and the Golgi-Cox assay were analyzed using Graph Pad prism software (GraphPad Software, San Diego, CA, USA) and a 2-way ANOVA with Bonferroni multiple testing. The error bars were calculated as standard error of the mean (SEM); *p*-values ≤ 0.05 were defined as significant. 

### 4.12. Data Availability

The raw MS-data are available at http://dx.doi.org/doi:10.20348/STOREDB/1132/1198.

## 5. Conclusions

In conclusion, the data from this study corroborated the results from the previous study regarding behavioral effects [12]. Both studies strongly suggested that a scenario of early postnatal exposure to a combination of ketamine and low-dose radiation, comparable to that found in clinical situations, was able to persistently induce cognitive impairment and changes in the neuronal structure. The neonatal window used in this study corresponded to the human brain developmental period that starts around the third trimester of pregnancy and expands over the first two years of life. Considering that ketamine is one of the most commonly used sedative agents in pediatric emergency departments [50], these results raise concern over the detrimental long-term effects on cognitive function. Whether the combination of ketamine and low-dose radiation is able to induce and exacerbate developmental neurobehavioral and cognitive defects in children should be investigated further, as this may be highly relevant for daily clinical practice.

## Figures and Tables

**Table 1:** Significantly deregulated proteins shared in the combined treatment groups with ketamine and irradiation.

	Symbol	Entrez Gene Name	Fold-Change		Biological Function	GO Number
			100 mGy Ket	200 mGy Ket		
1	ABHD10	abhydrolase domain containing 10	−2.014	−1.602	glucuronoside catabolic process	GO:0019391
2	ACAN	aggrecan	−2.742	−3.063	negative regulation of cell migration	GO:0030336
3	ADAM11	ADAM metallopeptidase domain 11	−1.344	−1.622	proteolysis	GO:0006508
4	ADAM23	ADAM metallopeptidase domain 23	−1.325	−1.409	proteolysis	GO:0006508
5	ARF6	ADP ribosylation factor 6	−1.432	−1.365	regulation of dendritic spine development	GO:0060998
6	ARMC1	armadillo repeat containing 1	−2.059	−1.752	metal ion transport	GO:0030001
7	ARPC1A	actin related protein 2/3 complex subunit 1A	1.358	1.515	regulation of actin filament polymerization	GO:0030833
8	ASPA	aspartoacylase	1.406	1.575	positive regulation of oligodendrocyte differentiation	GO:0048714
9	BRSK2	BR serine/threonine kinase 2	−1.680	−1.829	neuron differentiation	GO:0030182
10	CBR3	carbonyl reductase 3	−1.478	−1.577	cognition	GO:0050890
11	CDC42	cell division cycle 42	−1.588	−1.474	modification of synaptic structure	GO:0099563
12	CRK	CRK proto-oncogene. adaptor protein	−1.948	−2.240	dendrite development	GO:0016358
13	DNAJC6	DnaJ heat shock protein family (Hsp40) member C6	−1.381	−1.484	synaptic vesicle uncoating	GO:0016191
14	DYNLL2	dynein light chain LC8-type 2	−1.316	−1.329	microtubule-based process	GO:0007017
15	ELMO2	engulfment and cell motility 2	−2.612	−2.402	cytoskeleton organization	GO:0007010
16	FBXO2	F-box protein 2	−1.503	−1.307	regulation of protein ubiquitination	GO:0031396
17	GDPD1	glycerophosphodiester phosphodiesterase domain containing 1	−1.286	−1.293	N-acylethanolamine metabolic process	GO:0070291
18	GGT7	gamma-glutamyltransferase 7	−2.601	−4.805	regulation of response to oxidative stress	GO:1902883
19	GUK1	guanylate kinase 1	−1.496	−1.412	ATP metabolic process	GO:0046034
20	HIST1H2BD	histone cluster 1 H2B family member d	1.562	1.515	protein ubiquitination	GO:0016567
21	HNRNPUL1	heterogeneous nuclear ribonucleoprotein U like 1	−3.209	−1.488	RNA processing	GO:0006396
22	HTT	huntingtin	−1.862	−1.686	learning or memory	GO:0007611
23	IPO5	importin 5	−1.568	−1.478	protein import into nucleus	GO:0006606
24	MICU3	mitochondrial calcium uptake family member 3	−1.285	−1.484	mitochondrial calcium ion transmembrane transport	GO:0006851
25	NACA	nascent polypeptide associated complex subunit alpha	−1.297	−1.290	positive regulation of nucleic acid-templated transcription	GO:1903508
26	NDRG2	NDRG family member 2	−1.295	−1.326	nervous system development	GO:0001818
27	NIF3L1	NGG1 interacting factor 3 like 1	−1.649	−1.671	neuron differentiation	GO:0030182
28	NRP1	neuropilin 1	−1.935	−2.487	axon guidance	GO:0007411
29	OCIAD1	OCIA domain containing 1	1.529	1.818	regulation of stem cell differentiation	GO:2000736
30	PAK3	p21 (RAC1) activated kinase 3	−2.309	−2.063	dendritic spine development	GO:0060996
31	PCDH1	protocadherin 1	−1.874	−3.052	cell adhesion	GO:0007155
32	PFDN6	prefoldin subunit 6	−2.242	−1.669	protein folding	GO:0006457
33	PIP5K1C	phosphatidylinositol-4-phosphate 5-kinase type 1 gamma	−1.286	−1.306	axonogenesis	GO:0007409
34	PRKAR2A	protein kinase cAMP-dependent type II regulatory subunit alpha	−1.292	−1.351	modulation of chemical synaptic transmission	GO:0050804
35	PTGES3	prostaglandin E synthase 3	−1.543	−1.780	prostaglandin biosynthetic process	GO:0001516
36	PTPRS	protein tyrosine phosphatase. receptor type S	−1.545	−1.301	hippocampus development	GO:0021766
37	RAB1A	RAB1A. member RAS oncogene family	−1.302	−1.374	intracellular protein transport	GO:0006886
38	RAB5C	RAB5C. member RAS oncogene family	−1.323	−1.423	intracellular protein transport	GO:0006886
39	RABL6	RAB. member RAS oncogene family like 6	1.737	1.548	intracellular protein transport	GO:0006886
40	RIMBP2	RIMS binding protein 2	−1.385	−1.856	neuromuscular synaptic transmission	GO:0007274
41	RPLP2	ribosomal protein lateral stalk subunit P2	−1.543	−1.508	translational elongation	GO:0006414
42	SEC24C	SEC24 homolog C. COPII coat complex component	−1.342	−1.390	vesicle-mediated transport	GO:0016192
43	SLC1A4	solute carrier family 1 member 4	−1.414	−1.344	cognition	GO:0050890
44	SNCA	synuclein alpha	−1.432	−1.421	synaptic transmission	GO:0001963
45	STX7	syntaxin 7	−1.324	−1.298	vesicle-mediated transport	GO:0016192
46	SUCLG1	succinate-CoA ligase alpha subunit	1.342	1.356	succinyl-CoA metabolic process	GO:0006104
47	TIMM13	translocase of inner mitochondrial membrane 13	−1.845	−1.487	protein insertion into mitochondrial inner membrane	GO:0045039
48	TPD52	tumor protein D52	−1.468	−1.384	positive regulation of cell population proliferation	GO:0008284
49	TRAPPC10	trafficking protein particle complex 10	−1.392	−1.664	vesicle-mediated transport	GO:0016192
50	TRIO	trio Rho guanine nucleotide exchange factor	−1.562	−1.369	G-protein-coupled receptor signaling pathway	GO:0007186
51	TUBA8	tubulin alpha 8	−1.472	−1.343	microtubule cytoskeleton organization	GO:0000226
52	UBXN6	UBX domain protein 6	−1.360	−1.419	macroautophagy	GO:0016236
53	UCHL3	ubiquitin C-terminal hydrolase L3	−1.413	−1.427	adult walking behavior	GO:0007628
54	VBP1	VHL binding protein 1	−1.747	−1.809	protein folding	GO:0006457
55	WASF3	WAS protein family member 3	−1.904	−1.429	actin cytoskeleton organization	GO:0030036

## Supplementary Materials

Supplementary materials can be found at https://www.mdpi.com/1422-0067/20/23/6103/s1. Table S1. All quantified proteins with an identification based on at least two UP for the ketamine group. Table S2. All quantified proteins with an identification based on at least two UP for the 100 mGy group. Table S3. All quantified proteins with an identification based on at least two UP for the 200 mGy group. Table S4. All quantified proteins with an identification based on at least two UP for the 100 mGy Ket group. Table S5. All quantified proteins with an identification based on at least two UP for the 200 mGy Ket group. Figure S1. Ponceau staining and western blot bands. Figure S2. Representative hippocampal CA1 neurons for all experimental groups. Figure S3. Co-treatment with ketamine and irradiation does not affect the number of the apical dendrites, their branching or the spine density.
